# Mg Doped CuCrO_2_ as Efficient Hole Transport Layers for Organic and Perovskite Solar Cells

**DOI:** 10.3390/nano9091311

**Published:** 2019-09-13

**Authors:** Boya Zhang, Sampreetha Thampy, Wiley A. Dunlap-Shohl, Weijie Xu, Yangzi Zheng, Fong-Yi Cao, Yen-Ju Cheng, Anton V. Malko, David B. Mitzi, Julia W. P. Hsu

**Affiliations:** 1 Department of Materials Science and Engineering, The University of Texas at Dallas, Richardson, TX 75080, USA; 2 Department of Mechanical Engineering and Materials Science, Duke University, Durham, NC 27708, USA; 3 Department of Physics, The University of Texas at Dallas, Richardson, TX 75080, USA; 4 Department of Applied Chemistry, National Chiao Tung University, 1001 University Road, Hsinchu 30010, Taiwan

**Keywords:** Mg doped CuCrO_2_, hole transport layer, organic solar cells, perovskite solar cells

## Abstract

The electrical and optical properties of the hole transport layer (HTL) are critical for organic and halide perovskite solar cell (OSC and PSC, respectively) performance. In this work, we studied the effect of Mg doping on CuCrO_2_ (CCO) nanoparticles and their performance as HTLs in OSCs and PSCs. CCO and Mg doped CCO (Mg:CCO) nanoparticles were hydrothermally synthesized. The nanoparticles were characterized by various experimental techniques to study the effect of Mg doping on structural, chemical, morphological, optical, and electronic properties of CCO. We found that Mg doping increases work function and decreases particle size. We demonstrate CCO and Mg:CCO as efficient HTLs in a variety of OSCs, including the first demonstration of a non-fullerene acceptor bulk heterojunction, and CH_3_NH_3_PbI_3_ PSCs. A small improvement of average short-circuit current density with Mg doping was found in all systems.

## 1. Introduction

With continued increase in power conversion efficiency (PCE), organic and perovskite solar cells (OSCs and PSCs, respectively) are promising for low cost clean electricity generation [1,2,3]. Further enhancing the PCE of OSCs and PSCs requires not only the development of better absorber materials, but also suitable transport layer materials. In OSCs and PSCs, the absorber is sandwiched between an electron transport layer (ETL) and a hole transport layer (HTL), whose primary functions are to set up the built-in field across the absorber and selectively extract their respective carriers, while blocking the other type of carriers. In bulk heterojunction (BHJ) OSCs, the photogenerated excitons dissociate at the donor/acceptor interface to charged carriers. In PSCs, photoabsorption directly generates electrons and holes. These carriers then drift in opposite directions due to the built-in electric field, and travel through the transport layers to the electrodes [4]. Thus, both ETL and HTL play an important role in carrier extraction and device performance. For effective hole extraction from the absorber, the material used as HTL should possess good optical and electrical properties in addition to good physical and chemical stability. Extensive studies have been devoted to organic materials, such as poly(3,4-ethylenedioxythiophene) polystyrene sulfonate and 2,2′,7,7′-Tetrakis[*N*,*N*-di(4-methoxyphenyl)amino]-9,9′-spirobifluorene, as HTLs [5,6,7]. However, these materials are expensive and degrade under air exposure [8,9]. Alternatively, metal oxides are shown to be promising candidates for HTL due to their low cost and improved stability [10]. Commonly used metal oxides are MoO*_x_* and WO_3_ [11,12], but these *n*-type semiconductors do not block electrons [13,14]. Among *p*-type HTLs, NiO*_x_* has been shown to have promising performance [15,16]. However, it suffers from low conductivity and high visible light absorption [17,18]. Therefore, developing new inorganic *p*-type HTLs is crucial to achieve highly efficient and stable devices.

Delafossite (AMO_2_; A = Cu^1+^ or Ag^1+^ and M is a trivalent metal) compounds are *p*-type oxides and have drawn significant interest since Kawazoe et al. reported CuAlO_2_ as a transparent oxide with room temperature conductivity up to 1 S cm^−1^ [19]. Since then, many Cu-based delafossites have been synthesized with M = Al, Sc, Cr, Mn, Fe, Co, Ga, and Rh [20]. CuCrO_2_ (CCO) is particularly attractive due to its high conductivity [21]. Theoretical calculations and X-ray photoelectron spectroscopy (XPS) studies showed that Cu *d* states are dominant at the valence band maximum (VBM) and the intrinsic CCO conduction is through a Cu^I^/Cu^II^ mixed valence hole mechanism [22,23]. The size of CCO nanoparticles can be very small, ~10 nm [24,25]. In solar energy harvesting, it was first used in *p*-type dye sensitized solar cells (DSSCs) [25]. Moreover, CCO has been shown as a promising HTL in OSCs and PSCs [24,26,27,28,29]. To further increase the hole concentration, efforts have been made to replace the trivalent Cr^3+^ cation with a divalent dopant such as Ni^2+^, Mg^2+^, or Zn^2+^ [30,31,32]. In particular, Mg has been shown to be an excellent dopant to increase CCO conductivity [22,31,33]. Theoretical calculations showed that Mg doping induces low-formation energy defects just above the VBM in CCO and introduces new Cu *d* states in the bandgap, thus leading to a Cu^I^/Cu^II^ mixed valence and higher conductivity [22,33]. Compared with Be and Ca, defect states introduced by Mg are closest to the VBM, making it a more effective dopant [34]. Several experimental studies also confirm that Mg doping increases electrical conductivity [31,35,36,37]. In *p*-type DSSCs, Mg doped CCO (Mg:CCO) has performed superior to undoped CCO [38,39].

Based on our results of using undoped CCO as HTL in OSCs [24] and PSCs [27], we hypothesized that Mg:CCO could further improve the solar cell performance. Mg:CCO has not been applied as HTL in OSCs and, to our knowledge, there is only one report of using Mg:CCO as HTL in PSCs [40]. In this work, we examine the effect of Mg doping on CCO nanoparticles and their performance as HTLs in OSCs and PSCs. The CCO and Mg:CCO nanoparticles are synthesized by a hydrothermal method. The influence of Mg doping on structural, chemical, morphological, optical, and electronic properties of CCO films are carefully characterized by X-ray diffraction (XRD), energy-dispersive X-ray spectroscopy (EDX), transmission electron microscopy (TEM), dynamic light scattering (DLS), X-ray photoelectron spectroscopy (XPS), scanning electron microscopy (SEM), ultraviolet–visible (UV-vis) absorption spectrometry, photo-electron spectroscopy in air (PESA), and Kelvin probe (KP) techniques. Finally, spin-coated CCO and Mg:CCO nanoparticle films are used as HTLs in three different BHJ OSCs and methylammonium lead iodide (MAPbI_3_) PSCs. Time-resolved photoluminescence (TRPL) is applied to probe charge transfer between MAPbI_3_ and HTL, and XPS is used to examine elemental diffusion. This is the first work to apply Mg:CCO nanoparticle films as HTLs in OSCs and the first demonstration in a non-fullerene acceptor BHJ system.

## 2. Materials and Methods

The chemicals used in this study included copper(II) nitrate hemipentahydrate (Cu(NO_3_)_2_·2.5 H_2_O, Alfa Aesar, ACS, 98.0–102.0%, Tewksbury, MA, USA), chromium(III) nitrate nonahydrate (Cr(NO_3_)_3_·9 H_2_O, Alfa Aesar, 98.5%, Tewksbury, MA, USA), magnesium nitrate hydrate (Mg(NO_3_)_2_, Avocado Research Chemicals, 99.999%, London, United Kingdom), sodium hydroxide (NaOH, Sigma Aldrich, ≥97.0%, St. Louis, MO, USA), hydrochloric acid (HCl, Fisher Scientific, 37%, Houston, TX, USA), ethanol (EtOH, Fisher Chemical, anhydrous, Houston, TX, USA), 2-methoxyethanol (2-MOE, Acros Organics, 99+%, Houston, TX, USA), acetone (Fisher Chemical, certified ACS, Houston, TX, USA), 2-propanol (IPA, Fisher Chemical, certified ACS plus, Houston, TX, USA), poly(3-hexylthiophene-2,5-diyl) (P3HT, Rieke Metals, LLC, ≥96%, Lincoln, NE, USA), [6:6]-phenyl C61-butyric acid methyl ester (PC_61_BM, Solenne BV, >99%, Groningen, The Netherlands), [6,6]-phenyl C71-butyric acid methyl ester (PC_71_BM, Solenne BV, >99%, Groningen, The Netherlands), poly(5-bromo-4-(2-octyldodecyl)-selenophen2-yl)-5,6-difluorobenzothiadiazole-5,5′-bis-(trimethylstannyl)-2,2′-bithiophene (PFBT2Se2Th), poly[4,8-bis(5-(2-ethylhexyl)thiophen-2-yl)benzo[1,2-b;4,5-b’]dithiophene-2,6-diyl-alt-(4-(2-ethylhexyl)-3-fluorothieno[3,4-b]thiophene-)-2-carboxylate-2-6-diyl)] (PTB7-Th, Luminescence Technology Corp., New Taipei City, Taiwan), 3,9-bis(2-methylene-(3-(1,1-dicyanomethylene)-indanone))-5,5,11,11-tetrakis(4-hexylphenyl)-dithieno[2,3-d:2′,3′-d’]-s-indaceno[1,2-b:5,6-b’]dithiophene (ITIC, 1-Material Inc., Dorval, QC, Canada), chlorobenzene (CB, Sigma-Aldrich, anhydrous, 99.8%, St. Louis, MO, USA), 1,4-dichlorobenzene (DCB, Sigma Aldrich, anhydrous, 99%, St. Louis, MO, USA), diphenyl ether (DPE, Acros Organics, 99%, Houston, TX, USA), chloroform (CF, Sigma-Aldrich, ACS reagent, ≥99.8%, St. Louis, MO, USA), lead(II) iodide (TCI, 99.99%, Portland, OR, USA), methylammonium iodide (Dyesol, Queanbeyan, NSW, Australia), potassium iodide (Alfa Aesar, 99.998%, Tewksbury, MA, USA), dimethylformamide and dimethyl sulfoxide (DMF and DMSO, Sigma Aldrich, anhydrous grade, St. Louis, MO, USA), C_60_ (Luminescence Technology Corp., >99.5%, New Taipei City, Taiwan), and bathocuproine (BCP) (Sigma Aldrich, sublimed grade, 99.99%, St. Louis, MO, USA). All chemicals were used without further purification. PFBT2Se2Th was prepared according to previous publication, via copolymerization of 4,7-Bis(5-bromo-4-(2-octyldodecyl)selenophen-2-yl)-5,6-difluorobenzothiadiazole (FBT2Se) and 5,5′-bis(trimethylstannyl)-2,2′-bithiophene (2Th) [41].

### 2.1. CuCrO2 (CCO) and Mg:CCO Preparation

#### 2.1.1. Nanoparticle Synthesis

Mg:CCO nanoparticles with 0 at%, 5 at%, and 10 at% Mg doping levels were synthesized by a hydrothermal method as reported in previous literature [39]. First, 7.5 mmol Cu(NO_3_)_2_·2.5H_2_O and stoichiometric amounts of Cr(NO_3_)_3_·9H_2_O and Mg(NO_3_)_2_ were dissolved in 35 mL deionized (DI) water and stirred for 15 min at room temperature. Next, 2.5 g NaOH was added into the mixture and stirred for another 15 min at room temperature. The precursor solution was transferred into a 50 mL autoclave reactor (Col-Int Tech., Irmo, SC, USA), filled to 70% of its total volume. The hydrothermal reaction was carried out at 240 °C for CCO and 230 °C for Mg:CCO for 60 hours. Finally, the precipitate was washed using 2 M HCl and EtOH in sequence several times until the supernatant was colorless. After centrifuging, the mud was dried in a desiccator at room temperature overnight to obtain CCO or Mg:CCO powders.

#### 2.1.2. Suspension Preparation

CCO and Mg:CCO nanoparticles were dispersed into 2-MOE to make 2 mg mL^−1^ suspensions for materials characterization and fabrication of P3HT:PC_61_BM OSCs, PFBT2Se2Th:PC_71_BM OSCs, PTB7-Th:ITIC OSCs, and MAPbI_3_ PSCs. Just prior to film preparation, the suspensions were placed into a bath sonicator (Branson 3510, Plano, TX, USA) for 90 min, then filtered through a 1 μm PTFE filter (Thermos scientific, Titan3, Houston, TX, USA), re-sonicated for 90 min, and again filtered through a 0.45 μm PTFE filter (Biomed Scientific, Seattle, WA, USA).

#### 2.1.3. Film Preparation

2 mg mL^−1^ CCO and Mg:CCO suspensions were drop cast on gold-coated silicon substrates for EDX (Zeiss Supra 40) and XPS and on Cu grids (Ted Pella, Inc., Redding, CA, USA) for TEM imaging. CCO and Mg:CCO suspensions were spin coated multiple times on glass substrates for UV-vis (Ocean Optics USB 4000, Largo, FL, USA), spectroscopic ellipsometry (J. A. Woollam M-2000DI, Lincoln, NE, USA), and PESA (RKI Instruments Model AC-2, Union City, CA, USA), and on ITO substrates for SEM (Zeiss Supra 40, Lewisville, TX, USA) and KP (KP Technology SKP 5050, Caithness, Scotland) measurements and solar cells. After spin coating, the CCO and Mg:CCO films were annealed on a hot plate (120–175 °C) for 5 min in air to evaporate the solvent.

### 2.2. Materials Characterizaton

The crystalline phases of the nanoparticles were characterized by XRD using a Rigaku Ultima III diffractometer (The Woodlands, TX, USA) with Cu Kα (λ = 1.5418 Å) radiation. Powder diffraction files (PDFs) were used to identify characteristic peaks in the XRD patterns. Polytype compositions, crystal size and lattice parameters of XRD patterns were performed using Profex (an open source XRD and Rietveld refinement software, Solothurn, Switzerland) and structure files for phase identification were downloaded from the Crystallography Open Database (COD). The film morphologies were examined using SEM. The experimental Mg doping concentration was quantified using EDX. Nanoparticle size was measured from TEM images from the Delong LVEM5 Benchtop Electron Microscope (Delong TEM, Montreal, QC, Canada) equipped with the Q-Capture Pro 7 software. Hydrodynamic sizes were measured by DLS using a Malvern Zetasizer Nano ZS instrument (Malvern, United Kingdom). The lattice fringe distances were determined from high resolution TEM (HR TEM) images of nanoparticles obtained using a JEOL JEM2100 TEM (Peabody, MA, USA). The elemental compositions and chemical states of the films were analyzed by XPS, using a PHI 5000 Versa Probe II equipped with an Al Kα source and a hemispherical analyzer. XPS data were taken at a 45° takeoff angle with a pass energy of 23.5 eV. Optical transmission of the films was characterized by UV-vis over the wavelength range from 178 to 890 nm. The band gap energy was determined from Tauc plots of the UV-vis absorbance data. Film thickness was obtained by ellipsometry at 55°, 65°, and 75° incident angles over the wavelength range from 280 to 1690 nm. Ionization energy was measured from 4.7 to 5.8 eV with a 0.05 eV energy step using PESA with deuterium lamp intensity at 100 nW. The work function was measured using a KP apparatus (SKP5050, KP Technology) referenced to Au at 5.15 eV.

### 2.3. Solar Cell Fabrication and Testing

#### 2.3.1. P3HT:PC_61_BM OSCs

P3HT:PC_61_BM devices were fabricated on patterned ITO substrates (Xinyan Technology Ltd., Kwun Tong, Hong Kong, 15 Ω sq^−1^). The substrates were rinsed using soapy water, acetone, and IPA, followed by UV-ozone treatment for 20 min. 2 mg mL^−1^ CCO and Mg:CCO suspensions were spin coated at 2000 rpm for 30 s on top of the ITO substrates. The thickness of CCO and Mg:CCO nanoparticle films are ~18 nm; see Section 3.2 for details. 23 mg mL^−1^ P3HT and 23 mg mL^−1^ PC_61_BM were dissolved in CB and stirred at 50 °C overnight. The P3HT:PC_61_BM active layer (~200 nm thick) was made by dispensing 35 μL P3HT:PC_61_BM solution on a spinning substrate at 1200 rpm for 60 s, followed by annealing at 170 °C in N_2_ for 10 min. Finally, 7 nm Ca and 100 nm Al were sequentially evaporated on top of the active layer. The current-voltage (*J-V*) measurements were carried out using a 2635A Keithley low-noise sourcemeter under AM 1.5G 100 mW cm^−2^ illumination from a class AAA solar simulator (Abet Technologies) in a nitrogen filled glovebox. The diode area is 0.11 cm^2^ and the aperture area is 0.049 cm^2^.

#### 2.3.2. PFBT2Se2Th:PC_71_BM OSCs

PFBT2Se2Th:PC_71_BM devices were fabricated and tested similarly to the description in Section 2.3.1 unless otherwise noted. Six mg mL^−1^ PFBT2Se2Th and 12 mg mL^−1^ PC_71_BM were dissolved in DCB with 5 vol % DPE and stirred at 100 °C overnight. This solution and ITO/CCO or Mg:CCO substrates were preheated at 100 °C. The PFBT2Se2Th:PC_71_BM active layer (~120 nm thick) was made by first dispensing 50 μL of PFBT2Se2Th:PC_71_BM solution on the substrate and then immediately starting spinning at 1200 rpm for 60 s, followed by drying in a vacuum chamber for 2 min.

#### 2.3.3. PTB7-Th:ITIC OSCs

PTB7-Th:ITIC devices were fabricated and tested similarly to the description in Section 2.3.1 unless otherwise noted. PTB7-Th and ITIC were blended in a 1:1 weight ratio, dissolved in a mixed solution (CB with 3 vol% CF) at a total concentration of 20 mg mL^−1^ and stirred at room temperature overnight. The PTB7-Th:ITIC active layer (~80 nm thick) was made by first dispensing 40 μL PTB7-Th:ITIC solution on the substrate and then immediately starting spinning at 1250 rpm for 60 s.

#### 2.3.4. MAPbI_3_ PSCs

MAPbI_3_ PSCs were fabricated by spin coating the MAPbI_3_ layer (~450 nm thick) according to an antisolvent-washing recipe first described by Ahn et al. [42], and thereafter thermally evaporating a C_60_/BCP electron transport layer and then Ag electrodes. The details of the deposition of each of these layers are exactly as described in our previous report [27], except that the antisolvent wash during MAPbI_3_ deposition was carried out 11–12 s after starting the spin recipe. After fabrication, the devices were encapsulated with Ossila E131 UV-cure epoxy and a glass coverslip. *J.*–*V* curves were measured using a Keithley 2401 sourcemeter and an Oriel solar simulator. The lamp intensity was initially calibrated to 1 sun using a reference Si solar cell from Newport Corp., and maintained at that intensity during the measurements by a reference photodiode. The aperture area of the PSCs is 0.1 cm^2^, as defined by a shadow mask. (*J*,*V*) points were collected by sampling the current 1 s after the bias was applied. AC external quantum efficiency measurements were performed using an Enlitech QE-R instrument equipped with a monochromated Xe lamp optically chopped at 165 Hz, and without applied electrical bias. XPS, using a Kratos Analytical Axis Ultra spectrometer equipped with a monochromated Al Kα source, was performed on ITO/HTL/MAPbI_3_ films to determine whether diffusion of Cu, Cr, or Mg from the HTL resulted in detectable levels of these elements at the surface of the perovskite film. TRPL experiments comparing ITO/MAPbI_3_ and ITO/HTLs/MAPbI_3_ structures were performed using a microscope-based time-resolved system [43]. Samples were excited by 405 nm/120 fs optical pulses at 7.6 MHz repetition rate produced by doubling the fundamental frequency of the Mira 900 laser and followed by pulse-picking (1 out of 10 pulses) via the acousto-optical modulator (NEOS Technologies). Excitation of 1 µW was focused on the sample via 0.6 NA objective, which also ensured a high photon collection efficiency to obtain PL signals. The collected emission was passed through a spectrometer and directed either to a CCD camera for PL spectral analysis or to a sensitive photon detector (MicroPhoton Devices MPD 50) for the wavelength-dependent PL lifetime measurements. PL decay curves were collected via the time-correlated single photon counting performed on board of Pico300E photon counting hardware (PicoQuant GmbH). The overall time resolution was better than 200 ps.

## 3. Results and Discussion

### 3.1. Structural, Compositional, and Morphological Characterizations

**Figure 1:**
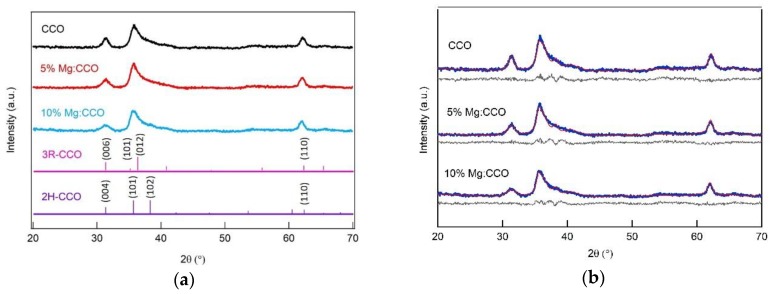
(**a**) X-ray diffraction (XRD) patterns of CuCrO_2_ (CCO) (black curve), 5% Mg:CCO (red curve), and 10% Mg:CCO (blue curve) powders. Two CCO polytypes, 3R-CCO (Powder diffraction files (PDF) #39-0247, pink sticks) and 2H-CCO (PDF#89-6743, purple sticks) are detected in all three XRD patterns. Prominent reflections are indexed between 30° to 40°, and ~62.0°. (**b**) Rietveld refinement of XRD patterns. The experimental (blue solid circle), calculated (red curve) and, difference (grey curve) patterns are shown. Structural files for the refinement are 3R-CCO (Crystallography Open Database (COD) No. 8104066) and 2H-CCO (COD No. 8104065).

Figure 1a shows XRD patterns of CCO (black curve), 5% Mg:CCO (red curve), and 10% Mg:CCO (blue curve) powders, respectively. XRD peaks are indexed as a mixture of two CCO polytypes, rhombohedral R3m (3R-CCO, pink sticks), and hexagonal P63/mmc (2H-CCO, purple sticks), for all three compounds. No impurity phases are detected. For the (110) reflection at ~62.0°, Mg doping results in a ~0.05° peak shift to the lower angle, indicating a larger lattice spacing. The (004) reflection at 31.4° exhibits broadening with Mg doping, indicating decreased crystal size along the *c* axis (Figure 1a and Table 1). Rietveld refinement was carried out in order to quantitatively determine the polytype composition, crystal size, and lattice parameters for each XRD pattern. Figure 1b shows the experimental (blue solid circle), calculated (red curve), and difference between experimental and calculated (grey curve) patterns for CCO, 5% Mg:CCO, and 10% Mg:CCO. Table 1 shows the results extracted from the Rietveld refinement. The polytype compositions for all three compounds are found to be ~60 ± 3% for 3R and ~40 ± 3% for 2H. The crystal sizes decrease monotonically from CCO to 10% Mg:CCO, from 7.8 to 4.5 nm for the 2H polytype calculated from the (004) reflection and from 9.6 to 8.7 nm for the 3R polytype calculated from the (110) reflection. However, the crystal size increases monotonically from 10.2 nm for CCO to 13.1 nm for 10% Mg:CCO in the (110) reflection for the 2H polytype. The tradeoff of the size changes for both polytypes leads to similar widths of the (110) reflection independent of Mg doping. Since delafossite nanocrystals often exhibit anisotropic morphology [25,27], it is reasonable that size changes differ for the (004) and (110) reflections. Similar orientation dependent size variation in Mg:CCO was reported by Bywalez et al. [35]. They attributed the decrease of crystal sizes along the *c* axis to Mg^2+^ obstructing growth of the delafossite crystal structure and stabilizing the spinel phase. However, there is no indication that this phase exists in our samples.

The lattice parameters for 3R-CCO and 2H–CCO polytypes were similar for CCO and 5% Mg:CCO. However, for 10% Mg:CCO, a larger *a* lattice parameter in both phases (3.00 Å versus 2.99 Å) and an increase in the *c* lattice parameter in the 2H–CCO phase (11.46 Å versus 11.43 Å) were observed. This lattice expansion was consistent with Mg substituting on the Cr site, rather than the Cu site [36], because the ionic radius of Mg^2+^ (0.72 Å) is larger than that of Cr^3+^ (0.62 Å) and smaller than that of Cu^+^ (0.77 Å). This result is consistent with the bond length increase between Cr and O sites after Mg doping predicted from theoretical calculations [33].

**Figure 2:**
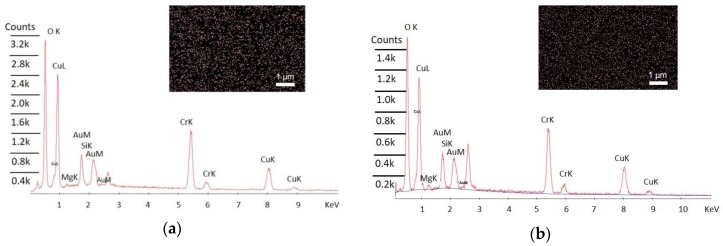
Energy-dispersive X-ray spectroscopy (EDX) spectra for (**a**) 5% Mg:CCO and (**b**) 10% Mg:CCO. Mappings of Mg element (inset) show uniform distribution.

In order to measure Mg concentration in CCO, EDX was performed on 5% Mg:CCO and 10% Mg:CCO. The insets of Figure 2a,b show that Mg is present and distributed uniformly in the Mg:CCO films. Mg/(Mg+Cr) represents the Mg concentration in the Mg:CCO films, which is calculated by atomic number effects (Z), absorption (A), and fluorescence (F) method from EDX spectra in Figure 2 [46]. Table 2 shows that the averaged Mg concentration in 5% Mg:CCO and 10% Mg:CCO measured from EDX is 4.0% and 9.8%, respectively. A possible Mg doping process is proposed similarly to the CCO formation mechanism as described by Miclau et al. [47]. During the hydrothermal synthesis of Mg:CCO nanoparticles, Cu^1+^, Cr^3+^, and Mg^2+^ ions can form ${\mathrm{Cu}(\mathrm{OH})}_{2}^{-}$, ${\mathrm{Cr}(\mathrm{OH})}_{4}^{-}$, and Mg(OH)_2_, respectively, at alkaline pH environment according to equations (1-3) below. Mg:CCO nanoparticles can then be formed from the metal hydroxides according to Equation (4) [48,49,50].The formation process of Mg:CCO nanoparticles are given in the following equations:

(1) $${\mathrm{Cu}}^{1+}{+2H}_{2}O\to {\mathrm{Cu}(\mathrm{OH})}_{2}^{-}{+2H}^{+}$$

(2) $${\mathrm{Cr}}^{3+}{+4H}_{2}O\to {\mathrm{Cr}(\mathrm{OH})}_{4}^{-}{+4H}^{+}$$

(3) $${\mathrm{Mg}}^{2+}{+2\mathrm{OH}}^{-}\to {\;\mathrm{Mg}(\mathrm{OH})}_{2}$$

(4) $${\mathrm{Cu}(\mathrm{OH})}_{2}^{-}{+(1-x)\cdot \mathrm{Cr}(\mathrm{OH})}_{4}^{-}{+x\cdot \mathrm{Mg}(\mathrm{OH})}_{2}{+2H}^{+}\to {\mathrm{CuCr}}_{1-x}{\mathrm{Mg}}_{x}O_{2}{+4H}_{2}{O-2x\cdot (\mathrm{OH})}^{-}$$

**Figure 3:**
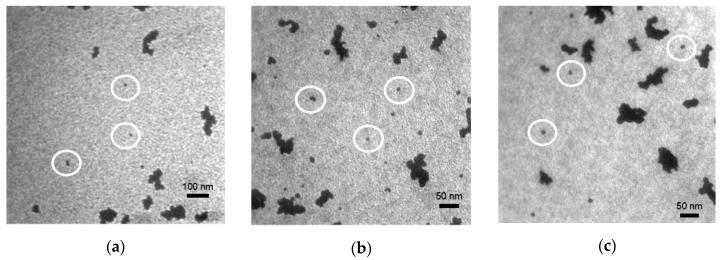
TEM images of (**a**) CCO, (**b**) 5% Mg:CCO, and (**c**) 10% Mg:CCO nanoparticles. Individual and double nanoparticles, as shown inside white circles in (**a**)–(**c**) are used to calculate size distributions shown in Table 2 and Figure 4a.

**Figure 4:**
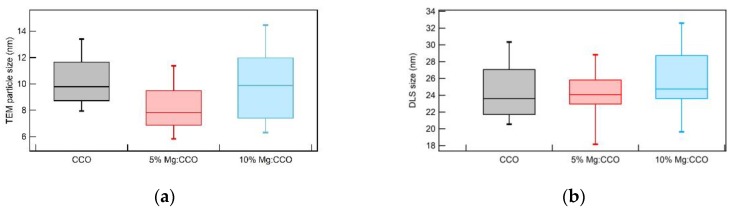
Box plots of (**a**) TEM-determined particle sizes from Figure 3 and (**b**) dynamic light scattering (DLS)-determined sizes for CCO (black color), 5% Mg:CCO (red color), and 10% Mg:CCO (blue color) nanoparticles. The percentiles are set to 90% whisker top, 75% box top, 25% box bottom, and 10% whisker bottom for each data set. In (**a**), ~50 individual nanoparticles are used in each data set. In (**b**), ~12 batches of DLS measurements are used in each data set.

TEM images of CCO (Figure 3a), 5% Mg:CCO (Figure 3b), and 10% Mg:CCO (Figure 3c) show the nanoparticles exist in individual or small clusters as well as large agglomerates. We only use individual or double nanoparticles (indicated by white circles) to determine particle sizes. The average nanoparticle size for CCO, 5% Mg:CCO, and 10% Mg:CCO is 10.3 ± 2.1, 8.2 ± 2.1, and 9.8 ± 3.0 nm, respectively. The size trend according to TEM results differs slightly from that of Rietveld-refined XRD data. One difference is that the particle size determined from XRD is analyzed for specific reflection and polytype (Table 1), while TEM images are two-dimensional projections of nanoparticles with random orientation. To examine the TEM size results in details, Figure 4a shows box plots of TEM particle sizes for CCO (black), 5% Mg:CCO (red), and 10% Mg:CCO (blue) nanoparticles. The ranges of CCO and 5% Mg:CCO nanoparticle sizes are smaller than that of 10% Mg:CCO nanoparticles. It is clear that 90% of the CCO nanoparticles are larger than 8 nm. In contrast, significant fractions of both types of Mg:CCO nanoparticles are between 6 and 8 nm. Thus, the statistics of TEM results show that Mg:CCO samples have greater numbers of smaller-sized particles, although 10% Mg doping appears to broaden the distribution. Considering the particle size results from XRD and TEM, overall Mg doping decreases CCO nanoparticle sizes because the XRD results show the size decreases along the *c* axis and TEM results show greater numbers of smaller sized Mg:CCO particles.

Figure 4b shows box plots of DLS sizes for CCO (black), 5% Mg:CCO (red), and 10% Mg:CCO (blue) nanoparticles dispersed in 2-MOE. For all nanoparticles, the 25–75% distributions are between 20 to 30 nm, indicating nanoparticles disperse well in 2-MOE. There are no significant differences among the three doping concentrations due to possibly similar hydrodynamic layer thickness. This is expected because the hydrodynamic layer is determined by solution ionic strength and hydrodynamic size and is often larger than dry particle size [51,52]. Since the Mg concentration in these nanoparticles is low, it is not surprising that hydrodynamic sizes for all samples are similar.

**Figure 5:**
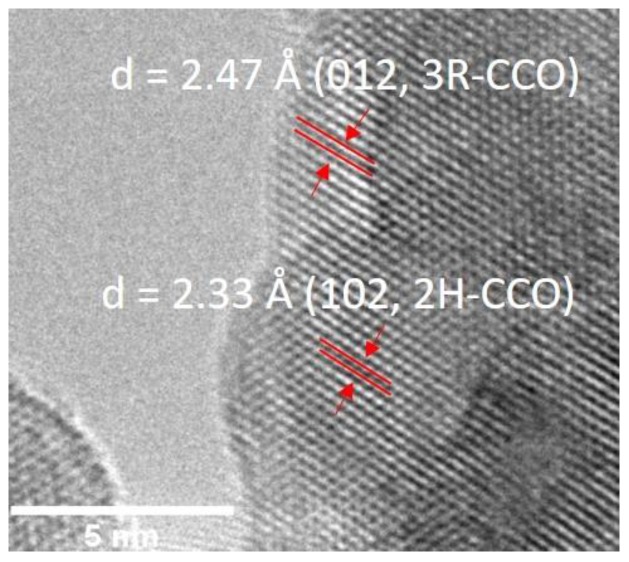
High resolution TEM (HR TEM) image of 5% Mg:CCO nanoparticle. Lattice spacings corresponding to the (012) reflection in 3R-CCO and the (102) reflection in 2H-CCO polytypes are indicated.

The HR TEM image of 5% Mg:CCO nanoparticles shows clear lattice fringes (Figure 5). The lattice spacing of 2.47 Å corresponds to the (012) reflection for 3R–CCO polytype. The lattice spacing of 2.33 Å corresponds to the (102) reflection for 2H–CCO polytype. No other lattice spacings corresponding to impurity phases are detected, consistent with XRD results.

**Figure 6:**
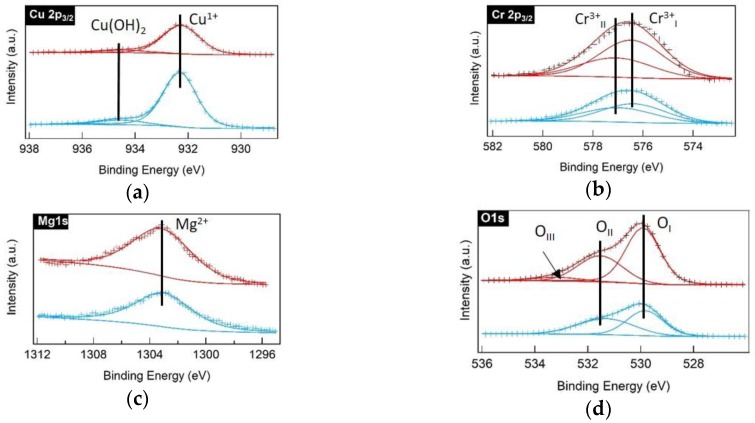
X-ray photoelectron spectroscopy (XPS) spectra of (**a**) Cu 2p_3/2_, (**b**) Cr 2p_3/2_, (**c**) Mg 1s, and (**d**) O 1s orbitals for 5% Mg:CCO (red) and 10% Mg:CCO (blue) nanoparticles. The measured XPS spectra are represented by cross symbols. The fitted XPS spectra are represented by solid curves. The black lines show the binding energies for Cu(OH)_2_, Cu^1+^, Cr^3+^_I_, Cr^3+^_II_, Mg^2+^, O_I_, O_II_, and O_III_. Cr^3+^_I_ represents Cr^3+^ as oxide, Cr^3+^_II_ is Cr^3+^ as hydroxide; O_I_ represents the lattice oxygen, O_II_ is hydroxyl species, and O_III_ is adsorbed water.

XPS studies were carried out in order to confirm the oxidation states of Cu, Cr, and Mg in the Mg:CCO powders. XPS data was analyzed using PHI Multipak software and peak fitting was done using a Gaussian–Lorentzian profile after a Shirley type background subtraction [53]. The binding energy was shifted using the valence band edge. The measured (cross symbol) and fitted (solid curve) XPS spectra of Cu 2p_3/2_, Cr 2p_3/2_, Mg 1s, and O 1s core levels for 5% Mg:CCO (red color) and 10% Mg:CCO (blue color) nanoparticles are shown in Figure 6. Deconvolution of the Cu 2p_3/2_ spectrum for both 5% and 10% Mg:CCO (Figure 6a) results in two peaks at 934.6 eV and 932.3 eV corresponding to binding energies of Cu(OH)_2_ and Cu^1+^, respectively, consistent with the literature [54,55]. The Cr 2p_3/2_ spectrum (Figure 6b) can be fitted to two peaks at 577.3 eV and 576.5 eV corresponding to binding energies of Cr^3+^ as hydroxide and Cr^3+^ as oxide, respectively [37,56]. These are similar to the binding energy peak positions of Cu^1+^ and Cr^3+^ oxide of undoped CCO nanoparticles reported in the literature [24]. Figure 6c shows the Mg 1s spectra, wherein the peak is located at binding energy of 1303.1 eV, corresponding to the Mg^2+^ oxidation state [39]. The O 1s spectrum (Figure 6d) shows peaks corresponding to lattice oxygen (O_I_) at 529.9 eV and hydroxyl groups (O_II_) at 531.5 eV for both 5% Mg:CCO and 10% Mg:CCO nanoparticles. A small-intensity peak at 533 eV corresponding to adsorbed water for 5% Mg:CCO nanoparticles is observed [54,57].

### 3.2. Optical and Electronic Characterizations

**Figure 7:**
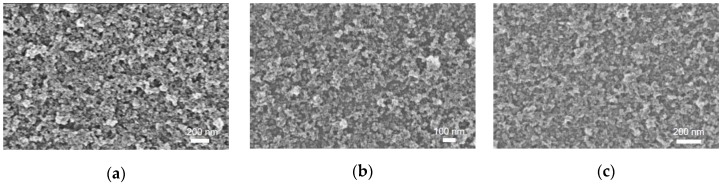
SEM images of (**a**) CCO, (**b**) 5% Mg:CCO, and (**c**) 10% Mg:CCO films on ITO substrates.

**Figure 8:**
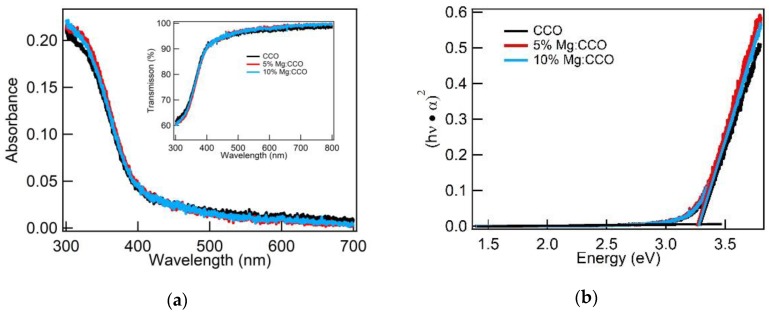
(**a**) Ultraviolet–visible (UV-vis) absorbance and transmission (inset) spectra and (**b**) Tauc plots and linear fits of the band edge (straight lines) for CCO (black curve), 5% Mg:CCO (red curve), and 10% Mg:CCO (blue curve) films.

The thickness of CCO and Mg:CCO nanoparticle films are controlled by the number of coating cycles that were performed during deposition [27]. Figure 7a–c shows SEM images for CCO, 5% Mg:CCO, and 10% Mg:CCO films; no regions of bare substrate are seen for all films. Figure 8a shows the UV-vis absorbance and transmission (inset) spectra of well-covered CCO (black), 5% Mg:CCO (red), and 10% Mg:CCO (blue) films. The absorbance values at 300 nm lie between 0.21 and 0.22 for all films, which are highly transparent (transmission > 90%) in the visible region. All three films are 18 nm thick as determined by ellipsometry. The direct band gap (*E_g_*) is extrapolated from the Tauc plot (Figure 8b). The average values of the direct *E_g_* are 3.27 ± 0.02 eV, 3.25 ± 0.03 eV, and 3.27 ± 0.03 eV for CCO, 5% Mg:CCO, and 10% Mg:CCO, respectively (Table 3). These values are the same within the uncertainty of the measurement (~0.03 eV). Thus, Mg doping does not affect the direct *E_g_* of CCO. The *E_g_* for pure CCO is consistent with our previous result [27].

**Figure 9:**
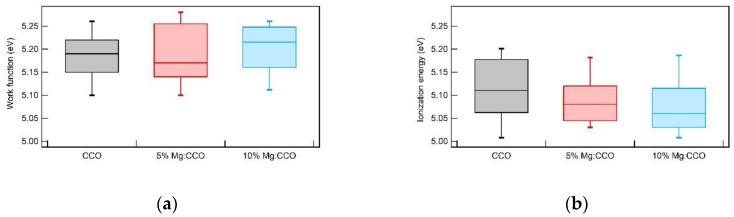
Box plots of (**a**) work function and (**b**) ionization energy for CCO (black color), 5% Mg:CCO (red color), and 10% Mg:CCO (blue color) films. The percentiles are set to 90% whisker top, 75% box top, 25% box bottom, and 10% whisker bottom for each data set. In (a), 20 batches of *WF* measurements are used in each data set. In (b), 13 batches of *IE* measurements are used in each data set.

Figure 9a shows box plots of the work function (*WF*) for CCO (black), 5% Mg:CCO (red), and 10% Mg:CCO (blue) films. The median *WF* values of CCO, 5% Mg:CCO, and 10% Mg:CCO films are 5.19, 5.17, and 5.22 eV, respectively. The 50^th^ percentile *WF* value for the 10% Mg:CCO is higher than that of CCO and 5% Mg:CCO. The 5% Mg:CCO films exhibit the largest spread with a long tail to the large *WF* than CCO films. Thus, Mg:CCO films generally appear to have higher *WF* values than CCO films, although the difference is below the level of statistical significance. Figure 9b shows box plots of the ionization energy (*IE*) for CCO (black), 5% Mg:CCO (red), and 10% Mg:CCO (blue) films. The median *IE* values of CCO, 5% Mg:CCO, and 10% Mg:CCO films are 5.11, 5.08, and 5.06 eV, respectively. The energy step for the *IE* measurement is 0.05 eV. The 50-percentile *IE* value decreases monotonically with increasing Mg concentration. Furthermore, the measured *IE* value is consistent with the *IE* of 5.1 eV from previous band structure calculations [27]. As shown in Figure 9, the overall *WF* values are larger than *IE* values, especially for Mg:CCO films. Thus, these films are *p*-type degenerately doped. The difference between *WF* and *IE* values (*WF – IE*) increases with Mg concentration from 0.08 eV for CCO to 0.16 eV for 10% Mg:CCO (Table 3), indicating that Mg:CCO films may have higher conductivity, consistent with previous results [35,36].

### 3.3. CCO and Mg:CCO as HTLs in OSCs and PSCs

**Figure 10:**
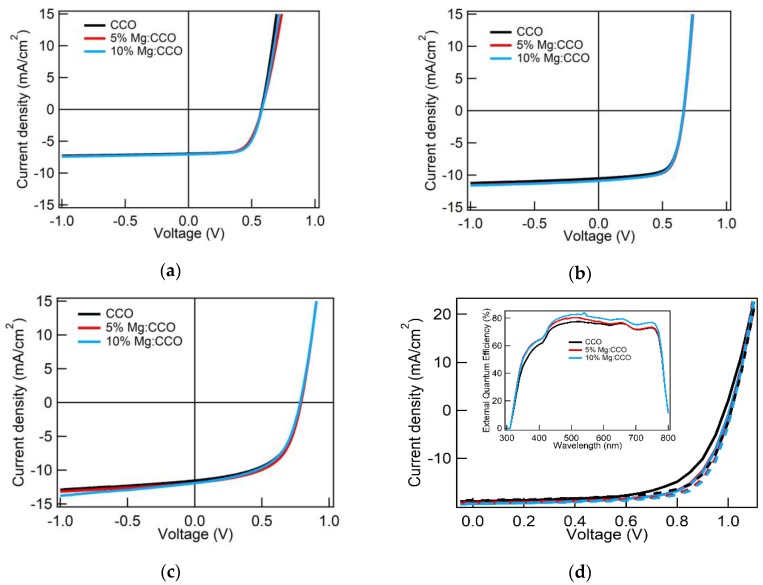
Average *J-V* curves (number of devices for each system is given in the footer of Table 4) of (**a**) P3HT:PC_61_BM OSCs, (**b**) PFBT2Se2Th:PC_71_BM OSCs, (**c**) PTB7-Th:ITIC OSCs, and (**d**) MAPbI_3_ PSCs measured in AM 1.5G 100 mW cm^−2^ illumination with CCO (black curve), 5% Mg:CCO (red curve), and 10% Mg:CCO (blue curve) hole transport layers (HTLs). In (d), solid *J-V* curves are measured under forward scan, dashed *J-V* curves are measured under reverse scan, and the inset is the external quantum efficiency (EQE) measurements of representative MAPbI_3_ cells with CCO (black curve), 5% Mg:CCO (red curve), and 10% Mg:CCO (blue curve) HTLs.

Figure 10a and Table 4 show the results of P3HT:PC_61_BM devices with CCO (black), 5% Mg:CCO (red), and 10% Mg:CCO (blue) HTLs. The average *J_sc_* of P3HT:PC_61_BM devices is higher with Mg:CCO HTL (from 6.94 mA cm^−2^ for CCO to ~7.05 mA cm^−2^ for Mg:CCO). The average *V_oc_* of Mg:CCO is also higher than that of undoped CCO HTL (~0.582 V versus 0.570 V). However, both Mg:CCO devices exhibit lower average *FF* (0.642 for 5% doping and 0.666 for 10% doping versus 0.685 for no doping). The tradeoff of the three parameters leads to similar *PCE* values for all devices independent of Mg doping. We note that the variation among different diodes is larger in *J_sc_* than *V_oc_* or *FF*, which is typical of OPV devices. Nonetheless, there is a systematic trend of average *J_sc_* increase with Mg doping.

Figure 10b and Table 4 show the results of PFBT2Se2Th:PC_71_BM devices with CCO (black), 5% Mg:CCO (red), and 10% Mg:CCO (blue) HTLs. The average *V_oc_* and *FF* are similar in all devices with values of ~0.665 V and ~0.685, respectively. The average *J_sc_* of the devices increases monotonically from 10.50 mA cm^−2^ for undoped CCO HTL to 10.88 mA cm^−2^ for 10% Mg:CCO HTL. Thus, the *PCE* values of PFBT2Se2Th:PC_71_BM devices are higher when Mg:CCO, instead of undoped CCO, is used as the HTL. Among different diodes, the variation of *J_sc_* is larger than that of *V_oc_* or *FF*. Moreover, Mg:CCO devices have even larger variation of *J_sc_*. However, a similar systematic trend of increasing average *J_sc_* as P3HT:PC_61_BM devices is observed in PFBT2Se2Th:PC_71_BM devices.

Figure 10c and Table 4 show the results of PTB7-Th:ITIC devices with CCO (black), 5% Mg:CCO (red), and 10% Mg:CCO (blue) HTLs. The average *J_sc_* of the devices increases monotonically from 11.55 mA cm^−2^ for undoped CCO HTL to 12.02 mA cm^−2^ for 10% Mg:CCO HTL. The average *V_oc_* and *FF* are highest for the 5% Mg:CCO in this batch of devices, but they do not depend on Mg doping in other batches. Generally, the *PCE* values of PTB7-Th:ITIC devices are higher when using Mg:CCO as the HTL due to the increase in *J_sc_*. This work is the first using CCO and Mg:CCO as HTL for BHJ OSCs with a non-fullerene acceptor.

Figure 10d and Table 4 show the results of MAPbI_3_ PSCs with CCO (black), 5% Mg:CCO (red), and 10% Mg:CCO (blue) HTLs under forward (solid lines) and reverse scans (dashed lines). Only slight hysteresis is seen, indicating minimal trap states at the CCO or Mg:CCO/perovskite interface. Under forward scan, the average *V_oc_* increases monotonically from 0.985 V for the undoped CCO HTL to 1.007 V for the 10% Mg:CCO HTL. Similar trends are observed in the *J_sc_* (from 18.91 mA cm^−2^ for the undoped CCO HTL to 19.40 mA cm^−2^ for the 10% Mg:CCO HTL) and *FF* (from 0.678 for the undoped CCO HTL to 0.703 for the 10% Mg:CCO HTL). Overall, the *PCE* of the devices improves monotonically from 12.64% for the undoped CCO HTL to 13.73% for the 10% Mg:CCO HTL. We note that the variation among different diodes is large for all the parameters. However, there are systematic trends of increases among the average *J_sc_*, *V_oc_*, and *FF* with Mg doping. Under reverse scan, the average *J_sc_* increases monotonically from 18.70 mA cm^−2^ for the undoped CCO HTL to 19.37 mA cm^−2^ for the 10% Mg:CCO HTL. The *FF* of the devices using the 5% Mg:CCO and 10% Mg:CCO HTLs were similar, 0.719, but for the undoped CCO HTL, a lower *FF* (0.697 versus 0.719) was observed. The *V_oc_* is similar in all devices with values of ~1.01 V. Overall, the *PCE* of the devices improves monotonically from 13.19% for the undoped CCO HTL to 14.12% for the 10% Mg:CCO HTL. As in the forward scan data, despite the variation among different diodes, there is a systematic trend of increasing average *J_sc_* with Mg doping. Jeong et al. observed that Mg:CCO produces PSCs with a slightly higher *J_sc_* and *V_oc_*, but a lower *FF*, resulting in no improvement in the *PCE*; however, they did not report Mg concentration [40]. The inset in Figure 8d shows the external quantum efficiency (EQE) at wavelength ranging from 300 to 800 nm for CCO (black), 5% Mg:CCO (red), and 10% Mg:CCO (blue) HTLs. A broadband increase in EQE is observed with Mg doping, consistent with the increases of average *J_sc_* in forward and reverse *J-V* scans.

**Figure 11:**
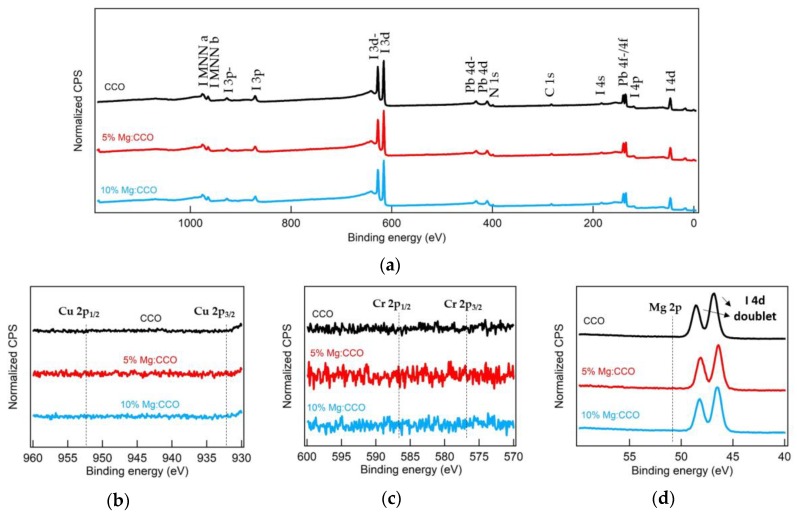
Normalized XPS (**a**) survey, (**b**) Cu 2p, (**c**) Cr 2p, and (**d**) Mg 2p spectra at the surfaces of MAPbI_3_ films on top of ITO/HTL. HTLs are CCO (black), 5% Mg:CCO (red), and 10% Mg:CCO (blue). In (**a**), all peaks are indexed by the component elements (C, N, Pb, and I) of MAPbI_3_. In (**b**–**d**), the dotted lines show binding energies for the Cu 2p, Cr 2p, and Mg 2p core levels of CCO and Mg:CCO. The positions of Cu 2p_1/2_, Cu 2p_3/2_, Cr 2p_1/2_, and Cr 2p_3/2_ peaks are indexed according to our previous CCO reports [24,27]. The position of Mg 2p peak is indexed according to the report from Hoogewijs et al. [65]. In (**d**), the peaks correspond to the I 4d orbitals; peaks due to the Mg 2p orbitals are not observed.

Several groups have reported elemental diffusion from inorganic transport layer into MAPbI_3_ when using CdS ETL [58], CrO*_x_* [59], and CuI [60] HTLs. In order to examine this possibility, we performed XPS studies on the surfaces of MAPbI_3_ films on top of ITO/HTL. Figure 11 shows the normalized XPS spectra of (a) survey, (b) Cu 2p, (c) Cr 2p, and (d) Mg 2p core levels for MAPbI_3_ films processed on top of CCO (black), 5% Mg:CCO (red), and 10% Mg:CCO (blue) HTLs. For all HTLs, all peaks in the survey spectra are indexed as the component elements (C, N, Pb, and I) of MAPbI_3_, consistent with our previous result [27]. The Cu 2p and Cr 2p spectral ranges are free of any peaks corresponding to Cu 2p or Cr 2p (dashed lines), indicating no presence of Cu and Cr elements at the surface of MAPbI_3_ layer. The Mg 2p spectrum shows two peaks at 48.2 eV and 46.6 eV corresponding to the binding energy of the I 4d orbitals [61]. No Mg 2p peak at 50.8 eV (dashed line) was detected, indicating no presence of Mg element. Thus, if metal diffusion from CCO and Mg:CCO HTLs into MAPbI_3_ occurs, it does so at a level below the sensitivity of XPS. This result is consistent with thermodynamic calculation: the calculated formation enthalpy of CCO is -6.0 eV [22], significantly lower compared to that of CdS (−1.5 eV) [62] and CuI (−0.3 eV) [63] and slightly lower compared to that of Cr_2_O_3_ (−5.9 eV) [64]. Mg:CCO has the same crystalline structure as CCO and the Mg doping content is small, thus, the formation enthalpy of Mg:CCO is expected to be similar to that of CCO. Thus, CCO and Mg:CCO are more stable and less likely to decompose or react than the aforementioned HTLs. Nevertheless, additional experimentation is warranted to explore the possibility of reactivity between CCO/Mg:CCO and perovskite phases.

**Figure 12:**
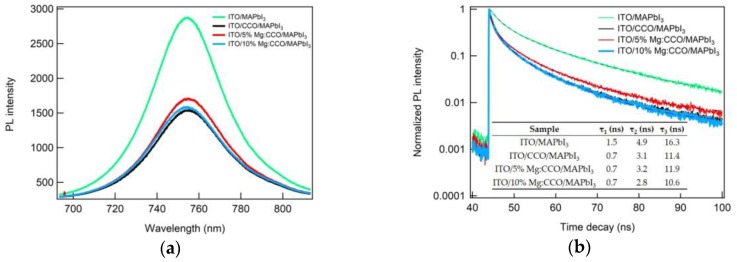
(**a**) Photoluminescence (PL) emission spectra and (**b**) time-resolved photoluminescence (TRPL) decay for ITO/MAPbI_3_ (green), ITO/CCO/MAPbI_3_ (black), ITO/5% Mg:CCO/MAPbI_3_ (red), and ITO/10% Mg:CCO/MAPbI_3_ (blue). In (**b**), the lines are fits to three exponential decays: dotted green line for ITO/MAPbI_3_, solid grey line for ITO/CCO/MAPbI_3_, dashed brown line for ITO/5% Mg:CCO/MAPbI_3_, and dotted-dashed blue line for ITO/10% Mg:CCO/MAPbI_3_. The inset table in (**b**) shows the fitted PL lifetimes for all samples.

In order to explore charge transport at the CCO and Mg:CCO/MAPbI_3_ interface, we performed TRPL measurements. Figure 12a shows the PL emission spectrum for ITO/MAPbI_3_ (green), ITO/CCO/MAPbI_3_ (black), ITO/5% Mg:CCO/MAPbI_3_ (red), and ITO/10% Mg:CCO/MAPbI_3_ (blue). For all samples, the main PL emission peaks are at ~750 nm, consistent with the literature [66]. PL intensities for MAPbI_3_ on top of CCO and Mg:CCO HTLs are lower compared to that of MAPbI_3_ on ITO, indicating CCO and Mg:CCO HTLs are effective in promoting charge transfer. However, PL intensities are similar among MAPbI_3_ on top of CCO and Mg:CCO HTLs. Figure 12b shows the normalized TRPL decay kinetics for ITO/MAPbI_3_ (green), ITO/CCO/MAPbI_3_ (black), ITO/5% Mg:CCO/MAPbI_3_ (red), and ITO/10% Mg:CCO/MAPbI_3_ (blue). With the addition of CCO and Mg:CCO HTLs, a faster PL decay is observed relative to ITO/MAPbI_3_. The inset table in Figure 12b shows the PL lifetimes extracted from three exponential fits in all samples (lines in Figure 12b). The *τ_1_*, *τ_2_*, and *τ_3_* lifetimes of the ITO/MAPbI_3_ structure are 1.5 ns, 4.9 ns, and 16.3 ns, respectively. After adding CCO and Mg:CCO HTLs, the *τ_1_*, *τ_2_*, and *τ_3_* decreases to 0.7 ns, ~3.0 ns, and ~11.0 ns, respectively, indicating enhanced charge extraction and consistent with the literature result [66]. Again, there are no significant differences in PL lifetimes among films of MAPbI_3_ on CCO and Mg:CCO HTLs. Mg doping in CCO is expected to lead lower PL intensity and shorter lifetimes. However, these effects are not discernable in our TRPL results, presumably because they may be confounded by factors besides charge transfer, such as surface recombination [67].

**Figure 13:**
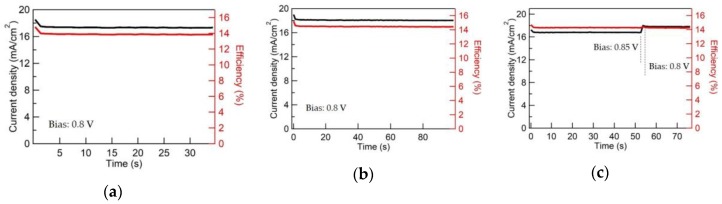
The stabilized photocurrents and efficiencies for the representative MAPbI_3_ cells with (**a**) CCO, (**b**) 5% Mg:CCO, and (**c**) 10% Mg:CCO HTLs. In (**a**,**b**), the applied bias is at 0.8 V. In (**c**), the applied bias is initially at 0.85 V. After 50 s, it switches to 0.8 V.

Figure 13 shows the stabilized photocurrents and efficiencies for representative MAPbI_3_ cells with CCO, 5% Mg:CCO, and 10% Mg:CCO HTLs. Time-dependent photocurrent measurements are taken at a bias of ~0.8 V with stabilized photocurrent values of 17.35 mA cm^−2^, 18.05 mA cm^−2^, and 17.81 mA cm^−2^ for CCO, 5% Mg:CCO, and 10% Mg:CCO HTLs, respectively. The values of the stabilized photocurrent are higher with Mg doping, reflecting the increases in *J_sc_* and *FF* observed in forward and reverse scans for Mg:CCO MAPbI_3_ PSCs. The stabilized efficiencies for CCO, 5% Mg:CCO, and 10% Mg:CCO HTLs are 13.89%, 14.43%, and 14.26%, respectively.

**Figure 14:**
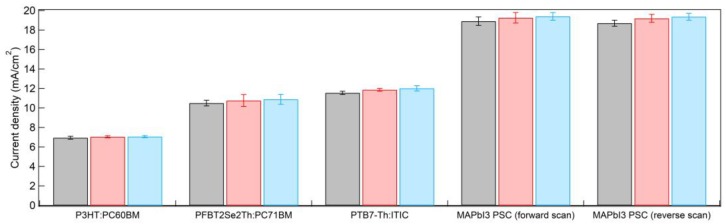
The average *J_sc_* barcharts with error bars for P3HT:PC_61_BM OSCs, PFBT2Se2Th:PC_71_BM OSCs, PTB7-Th:ITIC OSCs, and MAPbI_3_ PSCs under forward and reverse scans for CCO (black color), 5% Mg:CCO (red color), and 10% Mg:CCO (blue color) HTLs.

Figure 14 shows the bar charts of average *J_sc_* for P3HT:PC_61_BM OSCs, PFBT2Se2Th:PC_71_BM OSCs, PTB7-Th:ITIC OSCs, and MAPbI_3_ PSCs under forward and reverse scans for CCO (black color), 5% Mg:CCO (red color), and 10% Mg:CCO (blue color) HTLs. The average *J_sc_* of all OSCs and MAPbI_3_ PSCs are higher with Mg:CCO HTLs. The small average *J_sc_* increases in all systems may be partially attributed to the better conductivity of Mg:CCO HTLs resulting from the increased *WF* with respect to *IE* with Mg doping. Additionally, the broadband increase with Mg doping content in the EQE spectra of PSCs (Figure 10d inset) signifies that the increased HTL work function contributes to a stronger electric field within the device, more efficiently extracting photoexcited carriers regardless of the depth at which the generating photons are absorbed. If *V_oc_* and *FF* are independent of Mg doping, the *PCE* may be expected to increase due to the boost in *J_sc_*. However, they do not show a consistent trend from batch to batch. *V_oc_* and *FF* are more susceptible to film roughness, which can vary due to aggregation of the nanoparticles in the suspensions and variation in spin coating conditions. The tradeoff between *J_sc_* and *V_oc_*/*FF* results in little or no statistical *PCE* improvement (Table 4).

## 4. Conclusions

In summary, we synthesized CCO and Mg:CCO nanoparticles and successfully applied them as HTLs in OSCs and PSCs. Mg incorporation induces a slight lattice expansion by substituting larger ionic radii Mg^2+^ into the Cr^3+^ site. Rietveld refinement suggests that Mg doping decreases CCO nanoparticle size along the *c* axis but increases CCO nanoparticle size along the in-plane directions. Overall, both XRD and TEM results indicate that nanoparticle sizes are smaller with Mg doping. The average value of the direct *E_g_* is (3.26 ± 0.03) eV in all nanoparticle films. The *WF* values for all Mg concentrations are larger than the *IE* values, and their difference (*WF – IE*) increases with Mg concentration, consistent with increased *p*-type conductivity reported in the literature. OSCs and PSCs based on Mg:CCO HTLs show a consistent increase in average *J_sc_* in all four absorber systems despite large uncertainties; however, an overall enhancement in *PCE* is not clearly discernible (except in PSCs) due to different trends in other parameters and sample variation. No elemental (Cu, Cr, and Mg) diffusion from CCO and Mg:CCO HTLs is detected by XPS at the surface of MAPbI_3_ films. CCO and Mg:CCO HTLs effectively extract charge from the absorber, as evident in more PL quenching and shorter lifetimes when MAPbI_3_ is deposited on the HTLs. Mg doping in CCO HTLs enhances the stabilized efficiency for MAPbI_3_ PSCs. This work provides new insights related to the role that an Mg:CCO HTL may play in improving performance in a wide range of OSCs and MAPbI_3_ PSCs.

## Figures and Tables

**Table 1:** Rietveld refinement results for CCO, 5% Mg:CCO, and 10% Mg:CCO.

Sample	CCO	5% Mg:CCO	10% Mg:CCO
Polytype composition (%)	3R-CCO = 59.1 ± 3.0	3R-CCO = 59.5 ± 2.6	3R-CCO = 56.0 ± 2.7
	2H-CCO = 40.9 ± 3.0	2H-CCO = 40.5 ± 2.6	2H-CCO = 44.0 ± 2.7
*R_wp_* (%) ^1^	13.3	13.7	13.4
*R_exp_* (%) ^2^	9.0	9.0	9.1
*R_p_* (%) ^3^	9.0	10.9	10.5
*Χ^2^*	2.2	2.3	2.2
Crystal size (nm) based on (004)	7.8 ± 0.4 (2H-CCO)	5.6 ± 0.3 (2H-CCO)	4.5 ± 0.3 (2H-CCO)
Crystal size (nm) based on (110)	9.6 ± 0.9 (3R-CCO)	9.4 ± 0.9 (3R-CCO)	8.7 ± 1.0 (3R-CCO)
	10.2 ± 1.0 (2H-CCO)	12.3 ± 1.3 (2H-CCO)	13.1 ± 1.5 (2H-CCO)
Lattice parameter *a* ^4^ and *c* ^5^ (Å) for 3R-CCO	*a* = 2.99	*a* = 2.99	*a* = 3.00
	*c* =17.44	c =17.44	c =17.44
Lattice parameter *a* and *c* (Å) for 2H-CCO	*a* = 2.99	*a* = 2.99	*a* = 3.00
	*c* =11.43	c =11.44	c =11.46

^1^*R_wp_* is the weighted profile R-factor and the squared *R_wp_* is equal to the weighted sum of squared difference between the experimental and calculated intensity values over the weighted sum of squared experimental intensity values [44]. ^2^ *R_exp_* is the expected R-factor and the squared *R_exp_* is equal to the number of data points over the weighted sum of squared experimental intensity values [44]. ^3^ *R_p_* is the profile R-factor and is equal to the weighted sum of difference between the experimental and calculated intensity values over the weighted sum of experimental intensity values [45]. ^4 and 5^ *a* and *c* are the in-plane and out-of-lane lattice constants in the unit cell.

**Table 2:** Measured Mg doping concentration of CCO, 5% Mg:CCO, and 10% Mg:CCO films and average transmission electron microscopy (TEM) nanoparticle sizes of CCO, 5% Mg:CCO, and 10% Mg:CCO nanoparticles.

Sample	CCO	5% Mg:CCO	10% Mg:CCO
Mg/(Mg+Cr) (%) ^1^	0	4.0 ± 0.2	9.8 ± 1.3
Nanoparticle size (nm) ^2^	10.3 ± 2.1	8.2 ± 2.1	9.8 ± 3.0

^1^ Mg concentration is averaged over five EDX measurements. ^2^ Nanoparticle size is calculated from TEM images and mean size for each sample is averaged over 50 individual nanoparticles (Figure 3).

**Table 3:** Thickness and direct *E_g_* of CCO, 5% Mg:CCO, and 10% Mg:CCO films with ~0.22 absorbance at 300 nm wavelength.

Sample	Thickness (nm)	Direct *E_g_* (eV) ^1^	*WF_median_ – IE_median_* (eV)
CCO	18	3.27 ± 0.02	0.08
5% Mg:CCO	18	3.25 ± 0.03	0.09
10% Mg:CCO	18	3.27 ± 0.03	0.16

^1^ Direct *E_g_* is averaged over three measurements.

**Table 4:** The device parameters of OSCs and MAPbI_3_ PSCs with CCO, 5% Mg:CCO, and 10% Mg:CCO HTLs.

Device Type ^1^	HTL_Type	*J_sc_* (mA cm^−2^)	*V_oc_* (V)	*FF*	*PCE* (%)
P3HT:PC_61_BM	CCO	6.94± 0.15	0.570 ± 0.000	0.685 ± 0.008	2.71 ± 0.06
	5% Mg:CCO	7.04 ± 0.11	0.583 ± 0.005	0.642 ± 0.022	2.63 ± 0.08
	10% Mg:CCO	7.06 ± 0.11	0.581 ± 0.007	0.666 ± 0.017	2.73 ± 0.03
PFBT2Se2Th:PC_71_BM	CCO	10.50 ± 0.29	0.666 ± 0.007	0.684 ± 0.014	4.78 ± 0.18
	5% Mg:CCO	10.77 ± 0.61	0.664 ± 0.007	0.689 ± 0.011	4.93 ± 0.26
	10% Mg:CCO	10.88 ± 0.50	0.665 ± 0.007	0.678 ± 0.011	4.91 ± 0.27
PTB7-Th:ITIC	CCO	11.55 ± 0.17	0.786 ± 0.007	0.548 ± 0.010	4.97 ± 0.14
	5% Mg:CCO	11.87 ± 0.15	0.793 ± 0.005	0.559 ± 0.003	5.26 ± 0.08
	10% Mg:CCO	12.02 ± 0.27	0.785 ± 0.007	0.541 ± 0.011	5.11 ± 0.22
MAPbI_3_ PSC (forward scan)	CCO	18.91± 0.43	0.985 ± 0.058	0.678 ± 0.025	12.64 ± 0.99
	5% Mg:CCO	19.26 ± 0.54	1.003 ± 0.010	0.696 ± 0.023	13.45 ± 0.44
	10% Mg:CCO	19.40 ± 0.39	1.007 ± 0.014	0.703 ± 0.018	13.73 ± 0.34
MAPbI_3_ PSC (reverse scan)	CCO	18.70± 0.31	1.012 ± 0.006	0.697 ± 0.031	13.19 ± 0.71
	5% Mg:CCO	19.20 ± 0.41	1.011 ± 0.004	0.719 ± 0.012	13.96 ± 0.33
	10% Mg:CCO	19.37 ± 0.35	1.014 ± 0.006	0.719 ± 0.012	14.12 ± 0.28

^1^ For P3HT:PC_61_BM OSCs, 12, nine, and eight devices were measured for CCO, 5% Mg:CCO, and 10% Mg:CCO HTL, respectively. For PFBT2Se2Th:PC_71_BM OSCs, eight, seven, and 11 devices were measured for CCO, 5% Mg:CCO, and 10% Mg:CCO HTL, respectively. For PTB7-Th:ITIC OSCs, 10, eight, and nine devices were measured for CCO, 5% Mg:CCO, and 10% Mg:CCO HTL, respectively. For MAPbI_3_ PSCs, 10, 11, and 10 devices were measured for CCO, 5% Mg:CCO, and 10% Mg:CCO HTL, respectively, under both forward and reverse scans.
